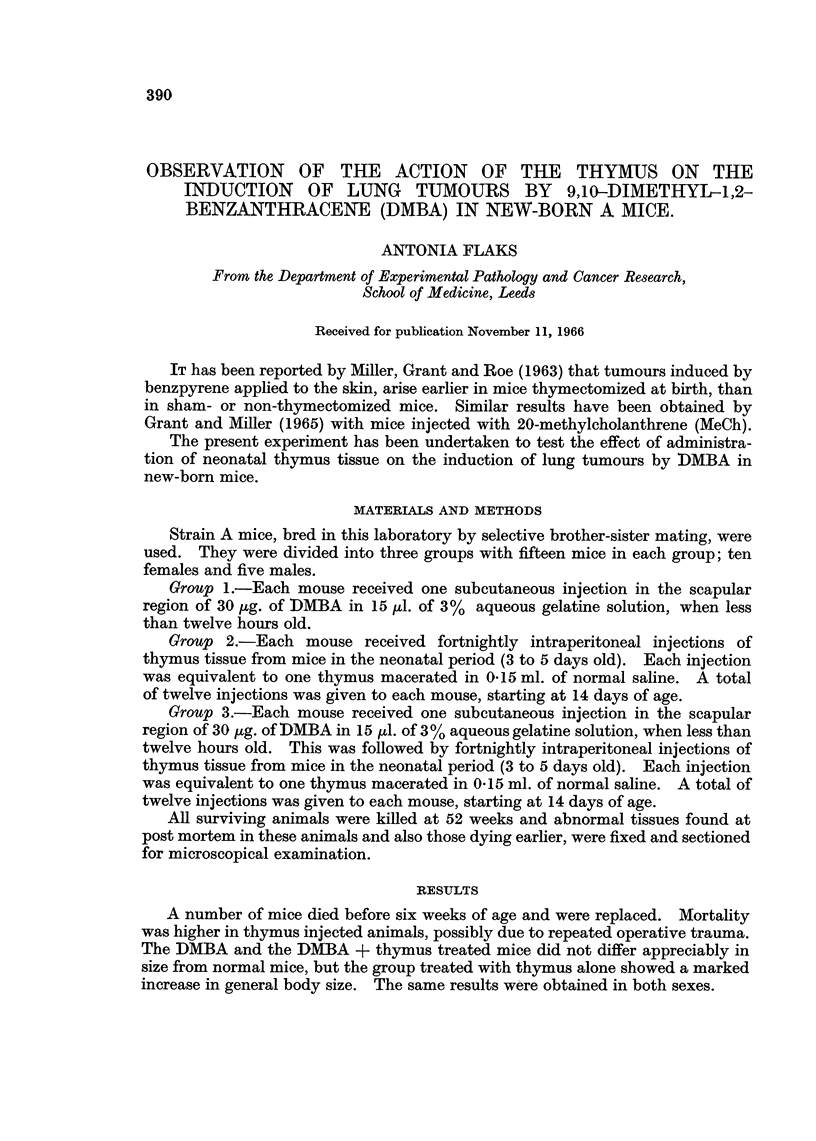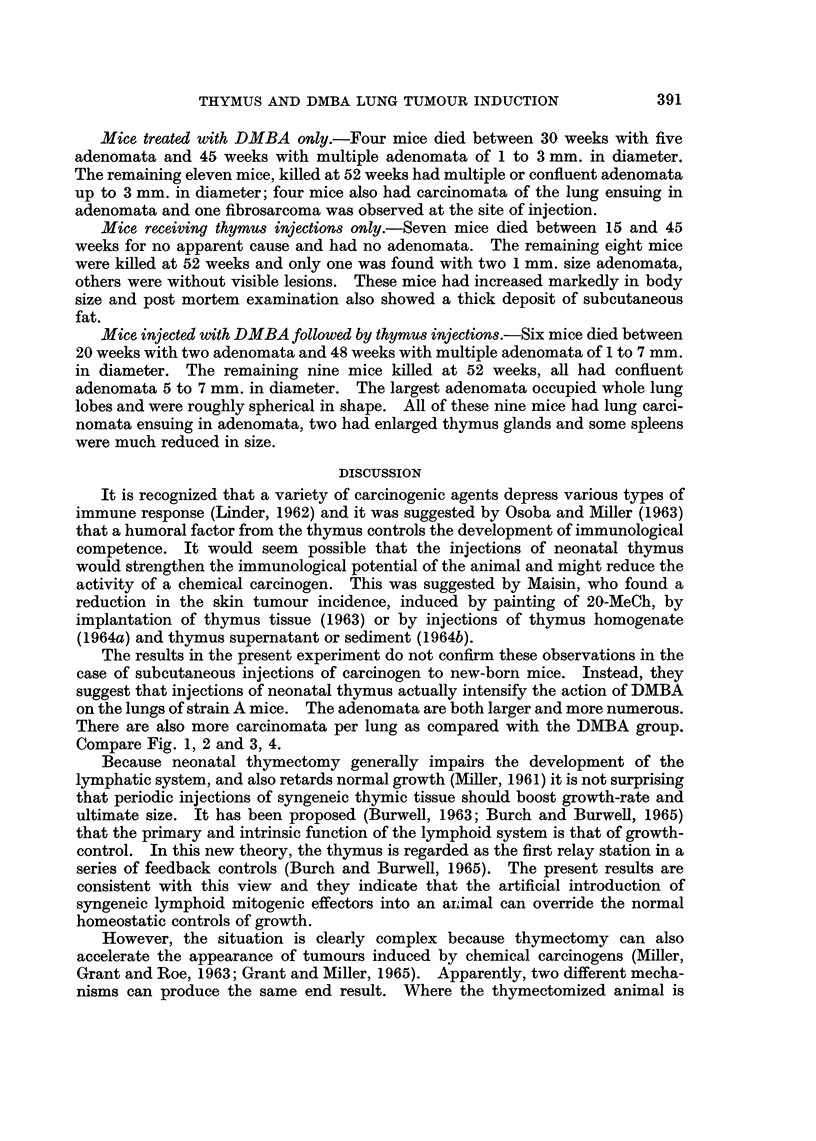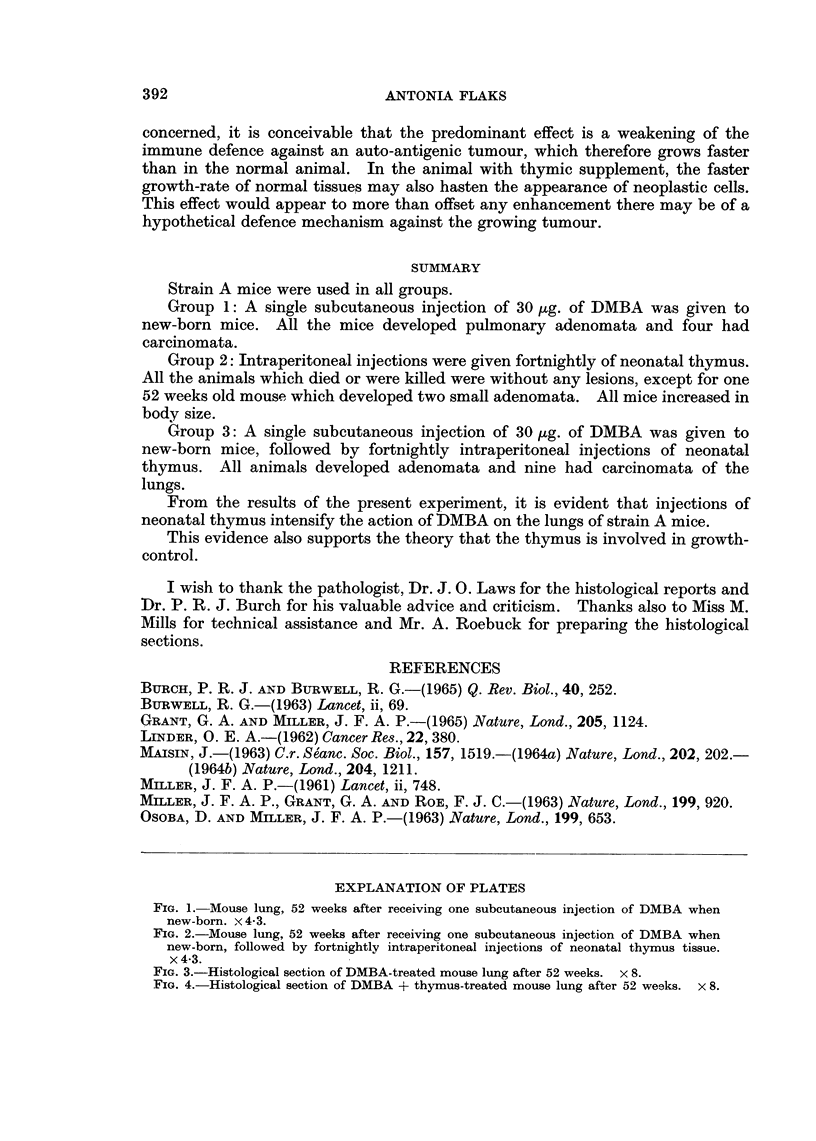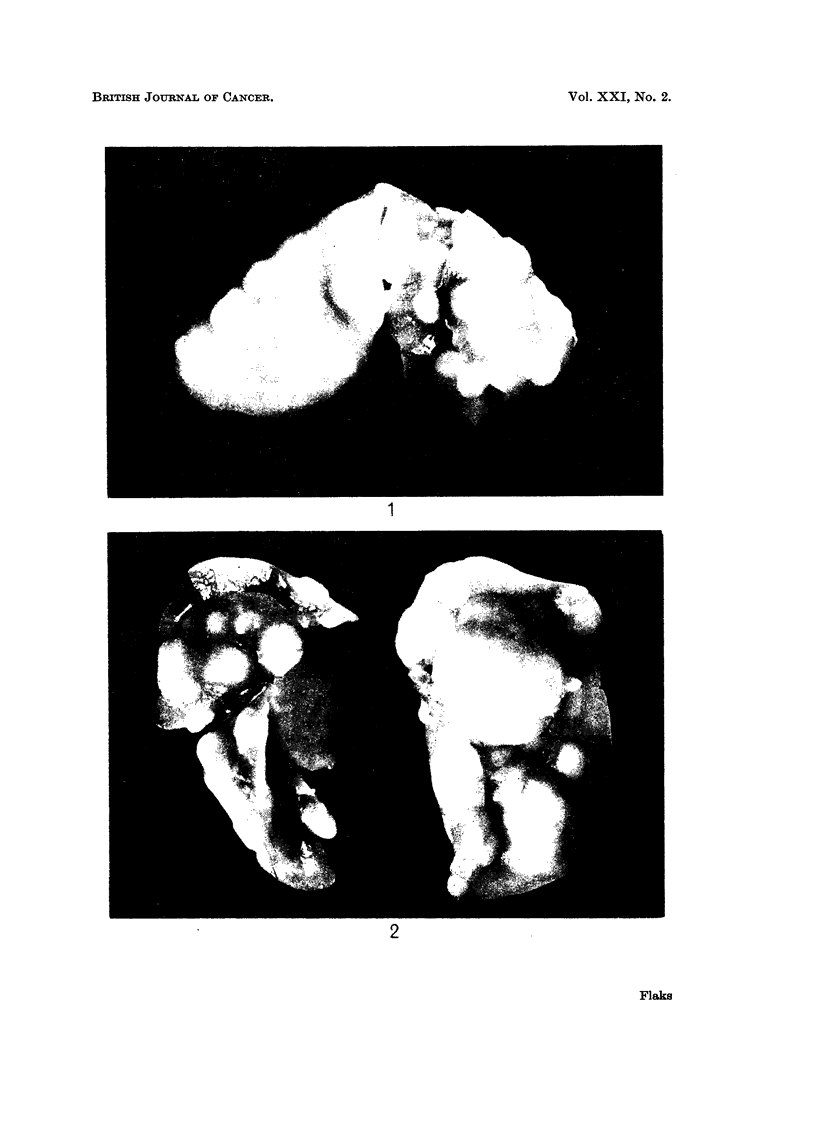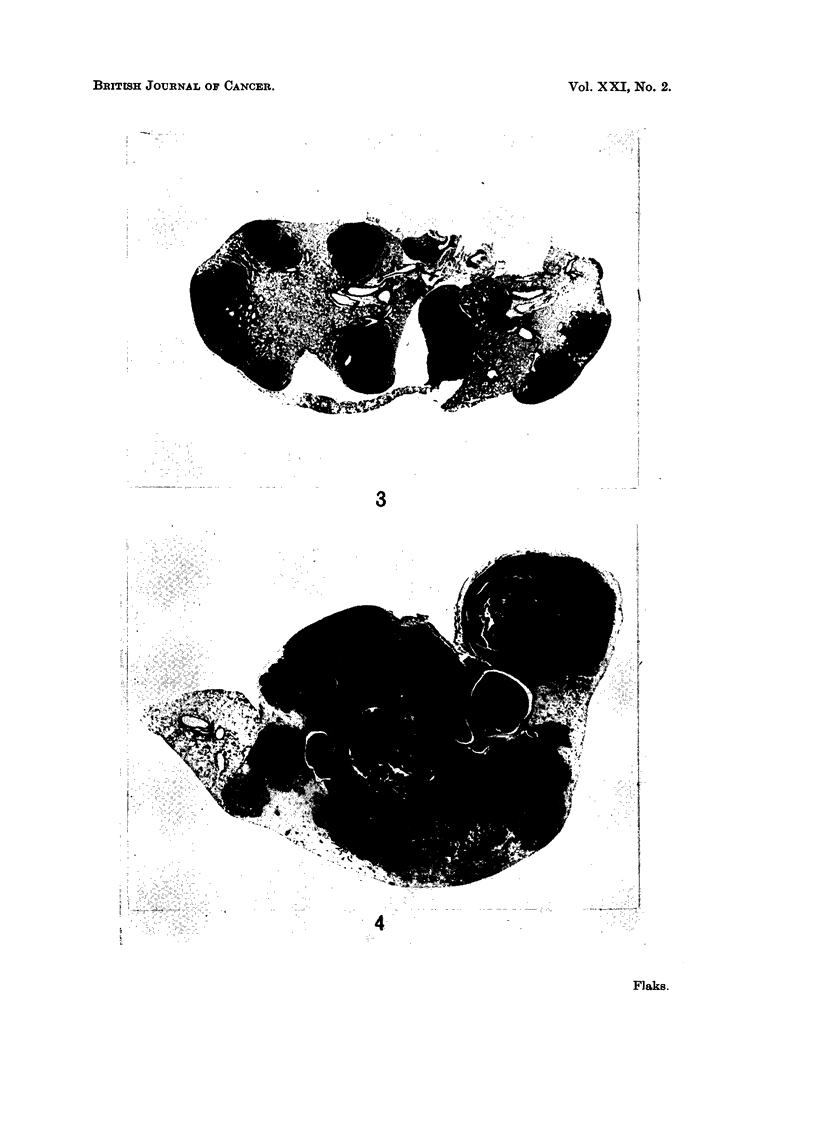# Observation of the action of the thymus on the induction of lung tumours by 9,10-dimethyl-1,2-benzanthracene (DMBA) in new-born A mice.

**DOI:** 10.1038/bjc.1967.44

**Published:** 1967-06

**Authors:** A. Flaks

## Abstract

**Images:**


### OBSERVATION OF THE ACTION OF THE THYMUS ON THE INDUCTION OF LUNG TUMOURS BY 9,10-DIMETHYL-1,2-BENZANTHRACENE (DMBA) IN NEW-BORN A MICE.

ANTONIA FLAKS

*From the Department of Experimental Pathology and Cancer Research, School of Medicine, Leeds*

Received for publication November 11, 1966

It has been reported by Miller, Grant and Roe (1963) that tumours induced by benzpyrene applied to the skin, arise earlier in mice thymectomized at birth, than in sham- or non-thymectomized mice. Similar results have been obtained by Grant and Miller (1965) with mice injected with 20-methylcholanthrene (MeCh).

The present experiment has been undertaken to test the effect of administration of neonatal thymus tissue on the induction of lung tumours by DMBA in new-born mice.

### MATERIALS AND METHODS

Strain A mice, bred in this laboratory by selective brother-sister mating, were used. They were divided into three groups with fifteen mice in each group; ten females and five males.

*Group* 1.—Each mouse received one subcutaneous injection in the scapular region of 30 $\mu$g. of DMBA in 15 $\mu$l. of 3% aqueous gelatine solution, when less than twelve hours old.

*Group* 2.—Each mouse received fortnightly intraperitoneal injections of thymus tissue from mice in the neonatal period (3 to 5 days old). Each injection was equivalent to one thymus macerated in 0·15 ml. of normal saline. A total of twelve injections was given to each mouse, starting at 14 days of age.

*Group* 3.—Each mouse received one subcutaneous injection in the scapular region of 30 $\mu$g. of DMBA in 15 $\mu$l. of 3% aqueous gelatine solution, when less than twelve hours old. This was followed by fortnightly intraperitoneal injections of thymus tissue from mice in the neonatal period (3 to 5 days old). Each injection was equivalent to one thymus macerated in 0·15 ml. of normal saline. A total of twelve injections was given to each mouse, starting at 14 days of age.

All surviving animals were killed at 52 weeks and abnormal tissues found at post mortem in these animals and also those dying earlier, were fixed and sectioned for microscopical examination.

### RESULTS

A number of mice died before six weeks of age and were replaced. Mortality was higher in thymus injected animals, possibly due to repeated operative trauma. The DMBA and the DMBA + thymus treated mice did not differ appreciably in size from normal mice, but the group treated with thymus alone showed a marked increase in general body size. The same results were obtained in both sexes.

*Mice treated with DMBA only.*—Four mice died between 30 weeks with five adenomata and 45 weeks with multiple adenomata of 1 to 3 mm. in diameter. The remaining eleven mice, killed at 52 weeks had multiple or confluent adenomata up to 3 mm. in diameter; four mice also had carcinomata of the lung ensuing in adenomata and one fibrosarcoma was observed at the site of injection.

*Mice receiving thymus injections only.*—Seven mice died between 15 and 45 weeks for no apparent cause and had no adenomata. The remaining eight mice were killed at 52 weeks and only one was found with two 1 mm. size adenomata, others were without visible lesions. These mice had increased markedly in body size and post mortem examination also showed a thick deposit of subcutaneous fat.

*Mice injected with DMBA followed by thymus injections.*—Six mice died between 20 weeks with two adenomata and 48 weeks with multiple adenomata of 1 to 7 mm. in diameter. The remaining nine mice killed at 52 weeks, all had confluent adenomata 5 to 7 mm. in diameter. The largest adenomata occupied whole lung lobes and were roughly spherical in shape. All of these nine mice had lung carcinomata ensuing in adenomata, two had enlarged thymus glands and some spleens were much reduced in size.

### DISCUSSION

It is recognized that a variety of carcinogenic agents depress various types of immune response (Linder, 1962) and it was suggested by Osoba and Miller (1963) that a humoral factor from the thymus controls the development of immunological competence. It would seem possible that the injections of neonatal thymus would strengthen the immunological potential of the animal and might reduce the activity of a chemical carcinogen. This was suggested by Maisin, who found a reduction in the skin tumour incidence, induced by painting of 20-MeCh, by implantation of thymus tissue (1963) or by injections of thymus homogenate (1964*a*) and thymus supernatant or sediment (1964*b*).

The results in the present experiment do not confirm these observations in the case of subcutaneous injections of carcinogen to new-born mice. Instead, they suggest that injections of neonatal thymus actually intensify the action of DMBA on the lungs of strain A mice. The adenomata are both larger and more numerous. There are also more carcinomata per lung as compared with the DMBA group. Compare Fig. 1, 2 and 3, 4.

Because neonatal thymectomy generally impairs the development of the lymphatic system, and also retards normal growth (Miller, 1961) it is not surprising that periodic injections of syngeneic thymic tissue should boost growth-rate and ultimate size. It has been proposed (Burwell, 1963; Burch and Burwell, 1965) that the primary and intrinsic function of the lymphoid system is that of growth-control. In this new theory, the thymus is regarded as the first relay station in a series of feedback controls (Burch and Burwell, 1965). The present results are consistent with this view and they indicate that the artificial introduction of syngeneic lymphoid mitogenic effectors into an animal can override the normal homeostatic controls of growth.

However, the situation is clearly complex because thymectomy can also accelerate the appearance of tumours induced by chemical carcinogens (Miller, Grant and Roe, 1963; Grant and Miller, 1965). Apparently, two different mechanisms can produce the same end result. Where the thymectomized animal is concerned, it is conceivable that the predominant effect is a weakening of the immune defence against an auto-antigenic tumour, which therefore grows faster than in the normal animal. In the animal with thymic supplement, the faster growth-rate of normal tissues may also hasten the appearance of neoplastic cells. This effect would appear to more than offset any enhancement there may be of a hypothetical defence mechanism against the growing tumour.

### SUMMARY

Strain A mice were used in all groups.

Group 1: A single subcutaneous injection of 30 μg. of DMBA was given to new-born mice. All the mice developed pulmonary adenomata and four had carcinomata.

Group 2: Intraperitoneal injections were given fortnightly of neonatal thymus. All the animals which died or were killed were without any lesions, except for one 52 weeks old mouse which developed two small adenomata. All mice increased in body size.

Group 3: A single subcutaneous injection of 30 μg. of DMBA was given to new-born mice, followed by fortnightly intraperitoneal injections of neonatal thymus. All animals developed adenomata and nine had carcinomata of the lungs.

From the results of the present experiment, it is evident that injections of neonatal thymus intensify the action of DMBA on the lungs of strain A mice.

This evidence also supports the theory that the thymus is involved in growth-control.

I wish to thank the pathologist, Dr. J. O. Laws for the histological reports and Dr. P. R. J. Burch for his valuable advice and criticism. Thanks also to Miss M. Mills for technical assistance and Mr. A. Roebuck for preparing the histological sections.

### REFERENCES

Burch, P. R. J. and Burwell, R. G.—(1965) *Q. Rev. Biol.*, **40**, 252.

Burwell, R. G.—(1963) *Lancet*, ii, 69.

Grant, G. A. and Miller, J. F. A. P.—(1965) *Nature, Lond.*, **205**, 1124.

Linder, O. E. A.—(1962) *Cancer Res.*, **22**, 380.

Maisin, J.—(1963) *C.r. Séanc. Soc. Biol.*, **157**, 1519.—(1964*a*) *Nature, Lond.*, **202**, 202.—(1964*b*) *Nature, Lond.*, **204**, 1211.

Miller, J. F. A. P.—(1961) *Lancet*, ii, 748.

Miller, J. F. A. P., Grant, G. A. and Roe, F. J. C.—(1963) *Nature, Lond.*, **199**, 920.

Osoba, D. and Miller, J. F. A. P.—(1963) *Nature, Lond.*, **199**, 653.

---

### EXPLANATION OF PLATES

Fig. 1.—Mouse lung, 52 weeks after receiving one subcutaneous injection of DMBA when new-born. ×4·3.

Fig. 2.—Mouse lung, 52 weeks after receiving one subcutaneous injection of DMBA when new-born, followed by fortnightly intraperitoneal injections of neonatal thymus tissue. ×4·3.

Fig. 3.—Histological section of DMBA-treated mouse lung after 52 weeks. ×8.

Fig. 4.—Histological section of DMBA + thymus-treated mouse lung after 52 weeks. ×8.